# Fluent genomics with *plyranges *and *tximeta*

**DOI:** 10.12688/f1000research.22259.1

**Published:** 2020-02-12

**Authors:** Stuart Lee, Michael Lawrence, Michael I. Love

**Affiliations:** 1 Econometrics and Business Statistics, Monash University, Clayton, Victoria, 3800, Australia; 2 Epigenetics and Development Division, Walter and Eliza Hall Institute of Medical Research, Parkville, Victoria, 3052, Australia; 3 Bioinformatics and Computational Biology, Genentech Inc, South San Fransisco, California, 94080, USA; 4 Department of Biostatistics, University of North Carolina at Chapel Hill, Chapel Hill, NC, 27516, USA; 5 Department of Genetics, University of North Carolina at Chapel Hill, Chapel Hill, NC, 27514, USA

**Keywords:** Gene Expression, Chromatin Accessibility, Workflow, Data Integration, Bioconductor, plyranges, tximeta

## Abstract

We construct a simple workflow for fluent genomics data analysis using the R/Bioconductor ecosystem. This involves three core steps: **import** the data into an appropriate abstraction, **model **the data with respect to the biological questions of interest, and **integrate** the results with respect to their underlying genomic coordinates. Here we show how to implement these steps to integrate published RNA-seq and ATAC-seq experiments on macrophage cell lines. Using *tximeta*, we **import** RNA-seq transcript quantifications into an analysis-ready data structure, called the *SummarizedExperiment*, that contains the ranges of the reference transcripts and metadata on their provenance. Using *SummarizedExperiment*s to represent the ATAC-seq and RNA-seq data, we **model **differentially accessible (DA) chromatin peaks and differentially expressed (DE) genes with existing Bioconductor packages. Using *plyranges* we then **integrate** the results to see if there is an enrichment of DA peaks near DE genes by finding overlaps and aggregating over log-fold change thresholds. The combination of these packages and their integration with the Bioconductor ecosystem provide a coherent framework for analysts to iteratively and reproducibly explore their biological data.

## Introduction

In this workflow, we examine a subset of the RNA-seq and ATAC-seq data from Alasoo *et al.* (2018), a study that involved treatment of macrophage cell lines from a number of human donors with interferon gamma (IFNg), *Salmonella* infection, or both treatments combined. Alasoo *et al.* (2018) examined gene expression and chromatin accessibility in a subset of 86 successfully differentiated induced pluripotent stem cells (iPSC) lines, and compared baseline and response with respect to chromatin accessibility and gene expression at specific quantitative trait loci (QTL). The authors found that many of the stimulus-specific expression QTL were already detectable as chromatin QTL in naive cells, and further hypothesize about the nature and role of transcription factors implicated in the response to stimulus.

We will perform a much simpler analysis than the one found in Alasoo *et al.* (2018), using their publicly available RNA-seq and ATAC-seq data (ignoring the genotypes). We will examine the effect of IFNg stimulation on gene expression and chromatin accessibility, and look to see if there is an enrichment of differentially accessible (DA) ATAC-seq peaks in the vicinity of differentially expressed (DE) genes. This is plausible, as the transcriptomic response to IFNg stimulation may be mediated through binding of regulatory proteins to accessible regions, and this binding may increase the accessibility of those regions such that it can be detected by ATAC-seq.

**Figure 1.**
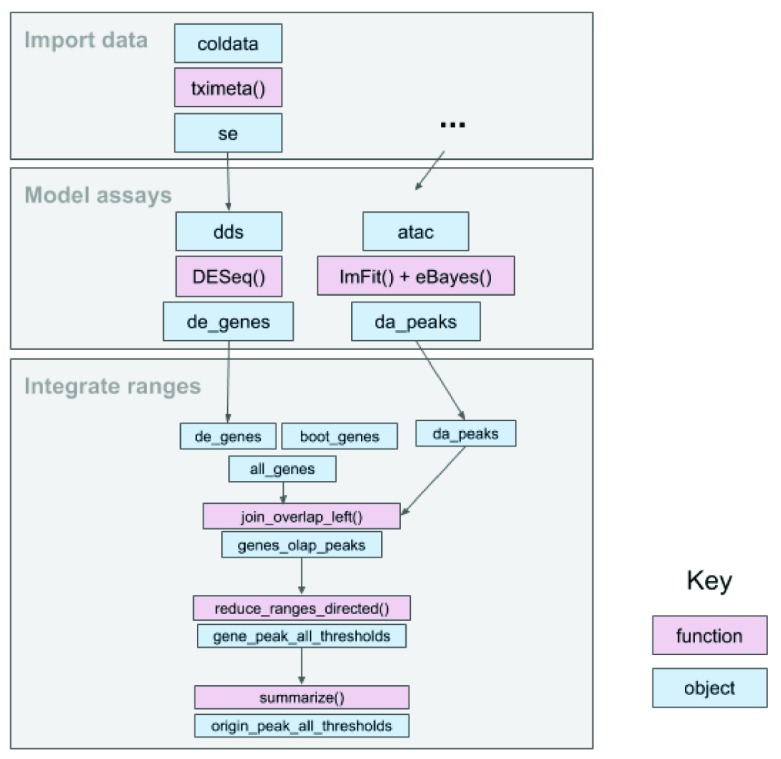
An overview of the fluent genomics workflow. First, we *import* data as a *SummarizedExperiment* object, which enables interoperability with downstream analysis packages. Then we *model* our assay data, using the existing Bioconductor packages *DESeq2* and *limma*. We take the results of our models for each assay with respect to their genomic coordinates, and *integrate* them. First, we compute the overlap between the results of each assay, then aggregate over the combined genomic regions, and finally summarize to compare enrichment for differentially expressed genes to non differentially expressed genes. The final output can be used for downstream visualization or further transformation.

Throughout the workflow ( Figure 1), we will use existing Bioconductor infrastructure to understand these datasets. In particular, we will emphasize the use of the Bioconductor packages *plyranges* and *tximeta*. The *plyranges* package fluently transforms data tied to genomic ranges using operations like shifting, window construction, overlap detection, etc. It is described by Lee *et al.* (2019) and leverages underlying core Bioconductor infrastructure ( Lawrence *et al.*, 2013; Huber *et al.*, 2015) and the *tidyverse* design principles Wickham *et al.* (2019).

The *tximeta* package described by Love *et al.* (2019) is used to read RNA-seq quantification data into R/Bioconductor, such that the transcript ranges and their provenance are automatically attached to the object containing expression values and differential expression results.

### Experimental data

The data used in this workflow is available from two packages: the *macrophage* Bioconductor ExperimentData package and from the workflow package *fluentGenomics* ( Lee & Love, 2020).

The *macrophage* package contains RNA-seq quantification from 24 RNA-seq samples, a subset of the RNA-seq samples generated and analyzed by Alasoo *et al.* (2018). The paired-end reads were quantified using *Salmon* ( Patro *et al.*, 2017), using the Gencode 29 human reference transcripts ( Frankish *et al.*, 2019). For more details on quantification, and the exact code used, consult the vignette of the macrophage package. The package also contains the Snakemake file that was used to distribute the *Salmon* quantification jobs on a cluster ( Köster & Rahmann, 2012).

The *fluentGenomics* package ( Lee & Love, 2020) contains functionality to download and generate a cached *SummarizedExperiment* object from the normalized ATAC-seq data provided by Alasoo & Gaffney (2017). This object contains all 145 ATAC-seq samples across all experimental conditions as analyzed by Alasoo *et al.* (2018). The data can be also be downloaded directly from the Zenodo deposition.

The following code loads the path to the cached data file, or if it is not present, will create the cache and generate a *SummarizedExperiment* using the the *BiocFileCache* package ( Shepherd & Morgan, 2019).


library(fluentGenomics)
path_to_se <-cache_atac_se()


We can then read the cached file and assign it to an object called atac.



atac <-readRDS(path_to_se)


A precise description of how we obtained this *SummarizedExperiment* object can be found in *Importing ATAC-seq data as a* *SummarizedExperiment object*.

## Import data as a *SummarizedExperiment*

### Using *tximeta* to import RNA-seq quantification data

First, we specify a directory dir, where the quantification files are stored. You could simply specify this directory with:



dir <-"/path/to/quant/files"


where the path is relative to your current R session. However, in this case we have distributed the files in the *macrophage* package. The relevant directory and associated files can be located using system.file.


dir <-system.file("extdata",package="macrophage")


Information about the experiment is contained in the coldata.csv file. We leverage the *dplyr* and *readr* packages (as part of the *tidyverse*) to read this file into R ( Wickham *et al.*, 2019). We will see later that *plyranges* extends these packages to accommodate genomic ranges.


library(dplyr)
##
## Attaching package: 'dplyr'
## The following objects are masked from 'package:stats':
##
##     filter, lag
## The following objects are masked from 'package:base':
##
##     intersect, setdiff, setequal, union





library(readr)
colfile <-file.path(dir,"coldata.csv")
coldata <-read_csv(colfile)%>%
dplyr::select(
names,
id =sample_id,
line =line_id,
condition =condition_name
)%>%
dplyr::mutate(
files =file.path(dir,"quants", names,"quant.sf.gz"),
line =factor(line),
condition =relevel(factor(condition),"naive")
)
## Parsed with column specification:
## cols(
##   names = col_character(),
##   sample_id = col_character(),
##   line_id = col_character(),
##   replicate = col_double(),
##   condition_name = col_character(),
##   macrophage_harvest = col_character(),
##   salmonella_date = col_character(),
##   ng_ul_mean = col_double(),
##   rna_extraction = col_character(),
##   rna_submit = col_character(),
##   library_pool = col_character(),
##   chemistry = col_character(),
##   rna_auto = col_double()
## )
coldata
## # A tibble: 24 x 5
##    names      id     line  condition  files
##    <chr>      <chr>  <fct> <fct>      <chr>
##  1 SAMEA1038~ diku_A diku~ naive      /Library/Frameworks/R.framework/Versions/~
##  2 SAMEA1038~ diku_B diku~ IFNg       /Library/Frameworks/R.framework/Versions/~
##  3 SAMEA1038~ diku_C diku~ SL1344     /Library/Frameworks/R.framework/Versions/~
##  4 SAMEA1038~ diku_D diku~ IFNg_SL13~ /Library/Frameworks/R.framework/Versions/~
##  5 SAMEA1038~ eiwy_A eiwy~ naive      /Library/Frameworks/R.framework/Versions/~
##  6 SAMEA1038~ eiwy_B eiwy~ IFNg       /Library/Frameworks/R.framework/Versions/~
##  7 SAMEA1038~ eiwy_C eiwy~ SL1344     /Library/Frameworks/R.framework/Versions/~
##  8 SAMEA1038~ eiwy_D eiwy~ IFNg_SL13~ /Library/Frameworks/R.framework/Versions/~
##  9 SAMEA1038~ fikt_A fikt~ naive      /Library/Frameworks/R.framework/Versions/~
## 10 SAMEA1038~ fikt_B fikt~ IFNg       /Library/Frameworks/R.framework/Versions/~
## # ... with 14 more rows


After we have read the coldata.csv file, we select relevant columns from this table, create a new column called files, and transform the existing line and condition columns into factors. In the case of condition, we specify the “naive” cell line as the reference level. The files column points to the quantifications for each observation – these files have been gzipped, but would typically not have the ‘gz’ ending if used from *Salmon* directly. One other thing to note is the use of the pipe operator, %>%, which can be read as “then”, i.e. first read the data, *then* select columns, *then* mutate them.

Now we have a table summarizing the experimental design and the locations of the quantifications. The following lines of code do a lot of work for the analyst: importing the RNA-seq quantification (dropping *inferential replicates* in this case), locating the relevant reference transcriptome, attaching the transcript ranges to the data, and fetching genome information. Inferential replicates are especially useful for performing transcript-level analysis, but here we will use a point estimate for the per-gene counts and perform gene-level analysis.

The result is a *SummarizedExperiment* object.



suppressPackageStartupMessages(library(SummarizedExperiment))
library(tximeta)
se <-tximeta(coldata,dropInfReps=TRUE)

## importing quantifications

## reading in files with read_tsv

## 1 2 3 4 5 6 7 8 9 10 11 12 13 14 15 16 17 18 19 20 21 22 23 24
## found matching linked transcriptome:
## [ GENCODE - Homo sapiens - release 29 ]
## loading existing TxDb created: 2019-11-22 01:02:58
## Loading required package: GenomicFeatures
## Loading required package: AnnotationDbi
##
## Attaching package: 'AnnotationDbi'
##
## The following object is masked from 'package:dplyr':
##
##     select
##
## loading existing transcript ranges created: 2019-11-22 01:06:45
## fetching genome info for GENCODE

se

## class: RangedSummarizedExperiment
## dim: 205870 24
## metadata(6): tximetaInfo quantInfo ... txomeInfo txdbInfo
## assays(3): counts abundance length
## rownames(205870): ENST00000456328.2 ENST00000450305.2 ...
##   ENST00000387460.2 ENST00000387461.2
## rowData names(3): tx_id gene_id tx_name
## colnames(24): SAMEA103885102 SAMEA103885347 ... SAMEA103885308
##   SAMEA103884949
## colData names(4): names id line condition


On a machine with a working internet connection, the above command works without any extra steps, as the tximeta function obtains any necessary metadata via FTP, unless it is already cached locally. The *tximeta* package can also be used without an internet connection, in this case the linked transcriptome can be created directly from a *Salmon* index and gtf.


makeLinkedTxome(
indexDir=file.path(dir,"gencode.v29_salmon_0.12.0"),
source="Gencode",
organism="Homo sapiens",
release="29",
genome="GRCh38",
fasta="ftp://ftp.ebi.ac.uk/pub/databases/gencode/Gencode_human/release_29/gencode.v29.transcripts.fa.gz",
gtf=file.path(dir,"gencode.v29.annotation.gtf.gz"),# local version
write=FALSE
)


Because *tximeta* knows the correct reference transcriptome, we can ask *tximeta* to summarize the transcript-level data to the gene level using the methods of Soneson *et al.* (2015).


gse <-summarizeToGene(se)

## loading existing TxDb created: 2019-11-22 01:02:58

## obtaining transcript-to-gene mapping from TxDb

## loading existing gene ranges created: 2019-11-23 02:30:13

## summarizing abundance

## summarizing counts

## summarizing length



One final note is that the start of positive strand genes and the end of negative strand genes is now dictated by the genomic extent of the isoforms of the gene (so the start and end of the reduced *GRanges*). Another alternative would be to either operate on transcript abundance, and perform differential analysis on transcript (and so avoid defining the TSS of a set of isoforms), or to use gene-level summarized expression but to pick the most representative TSS based on isoform expression.

### Importing ATAC-seq data as a *SummarizedExperiment* object

The *SummarizedExperiment* object containing ATAC-seq peaks can be created from the following tab-delimited files from Alasoo & Gaffney (2017):

- The sample metadata: ATAC_sample_metadata.txt.gz (<1M)
- The matrix of normalized read counts: ATAC_cqn_matrix.txt.gz (109M)
- The annotated peaks: ATAC_peak_metadata.txt.gz (5.6M)

To begin, we read in the sample metadata, following similar steps to those we used to generate the coldata table for the RNA-seq experiment:


atac_coldata <-read_tsv("ATAC_sample_metadata.txt.gz")%>%
select(
sample_id,
donor,
condition =condition_name
)%>%
mutate(condition =relevel(factor(condition),"naive"))


The ATAC-seq counts have already been normalized with *cqn* ( Hansen *et al.*, 2012) and log2 transformed. Loading the *cqn*-normalized matrix of log2 transformed read counts takes ~30 seconds and loads an object of ~370 Mb. We set the column names so that the first column contains the rownames of the matrix, and the remaining columns are the sample identities from the atac_coldata object.


atac_mat <-read_tsv("ATAC_cqn_matrix.txt.gz",
skip =1,
col_names =c("rownames", atac_coldata[["sample_id"]]))
rownames <- atac_mat[["rownames"]]
atac_mat <-as.matrix(atac_mat[,-1])
rownames(atac_mat) <- rownames


We read in the peak metadata (locations in the genome), and convert it to a *GRanges* object. The as_granges() function automatically converts the *data.frame* into a *GRanges* object. From that result, we extract the peak_id column and set the genome information to the build “GRCh38”. We know this from the Zenodo entry.


library(plyranges)
peaks_df <-read_tsv("ATAC_peak_metadata.txt.gz",
col_types =c("cidciicdc")
)

peaks_gr <- peaks_df%>%
as_granges(seqnames =chr)%>%
select(peak_id=gene_id)%>%
set_genome_info(genome ="GRCh38")


Finally, we construct a *SummarizedExperiment* object. We place the matrix into the assays slot as a named list, the annotated peaks into the row-wise ranges slot, and the sample metadata into the column-wise data slot:


atac <-SummarizedExperiment(assays =list(cqndata=atac_mat),
rowRanges=peaks_gr,
colData=atac_coldata)


## Model assays

### RNA-seq differential gene expression analysis

We can easily run a differential expression analysis with *DESeq2* using the following code chunks ( Love *et al.*, 2014). The design formula indicates that we want to control for the donor baselines ( line) and test for differences in gene expression on the condition. For a more comprehensive discussion of DE workflows in Bioconductor see Love *et al.* (2016) and Law *et al.* (2018).


library(DESeq2)
dds <-DESeqDataSet(gse,~line+condition)

## using counts and average transcript lengths from tximeta

# filter out lowly expressed genes
# at least 10 counts in at least 6 samples

keep <-rowSums(counts(dds)>=10) 
                        >=6
dds <- dds[keep,]


The model is fit with the following line of code:


dds <-DESeq(dds)
## estimating size factors
## using 'avgTxLength' from assays(dds), correcting for library size
## estimating dispersions
## gene-wise dispersion estimates
## mean-dispersion relationship
## final dispersion estimates
## fitting model and testing


Below we set the contrast on the condition variable, indicating we are estimating the log _2_ fold change (LFC) of IFNg stimulated cell lines against naive cell lines. We are interested in LFCs greater than 1 at a nominal false discovery rate (FDR) of 1%.


res <- results(dds,
contrast=c("condition","IFNg","naive"),
lfcThreshold=1,alpha=0.01)


**Figure 2.**
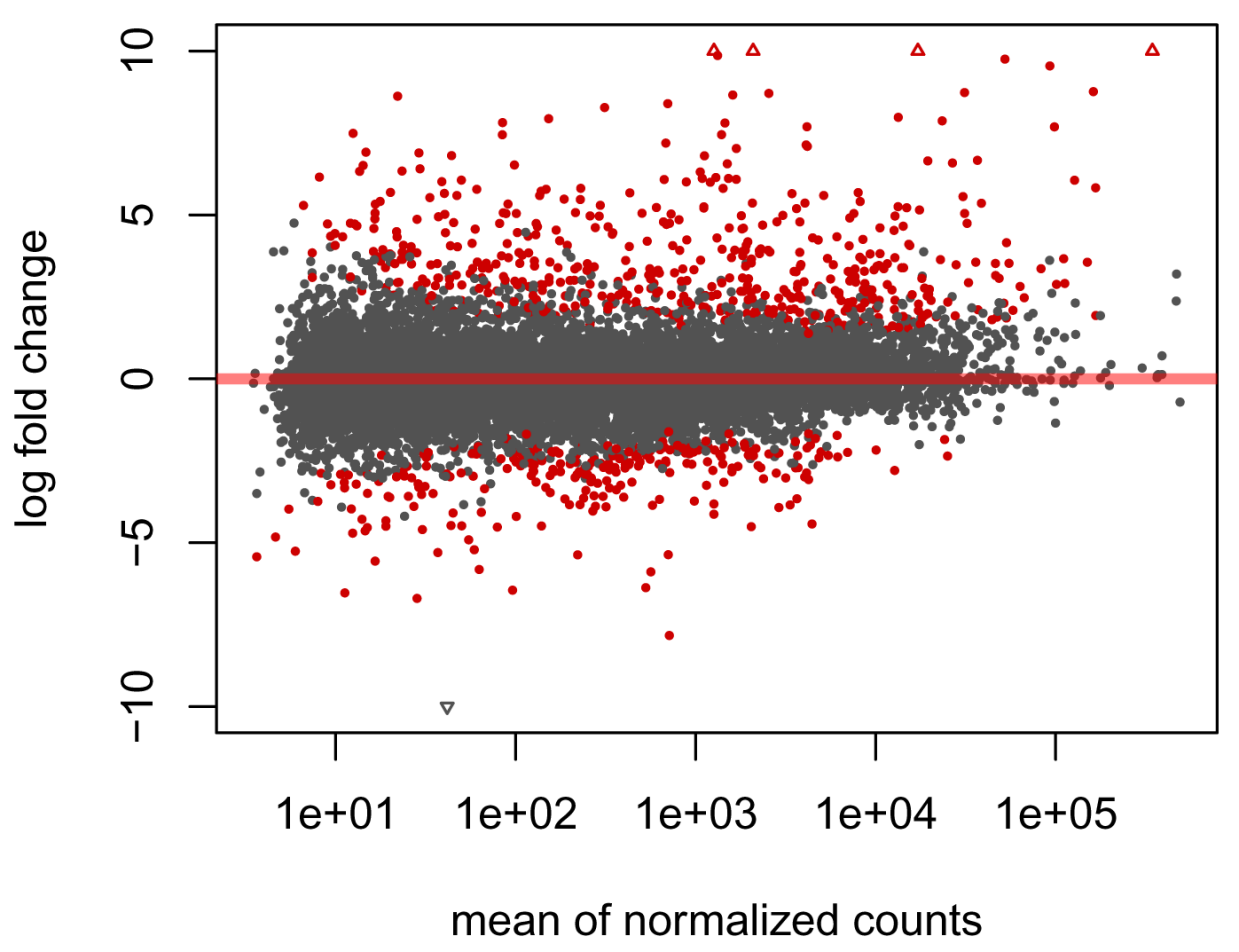
Visualization of *DESeq2* results as an “MA plot”. Genes that have an adjusted *p-value* below 0.01 are colored red.

To see the results of the expression analysis, we can generate a summary table and an MA plot ( Figure 2):


summary(res)

                        ##
## out of 17806 with nonzero total read count
## adjusted p-value < 0.01
## LFC > 1.00 (up)    : 502, 2.8%
## LFC < -1.00 (down) : 247, 1.4%
## outliers [1]       : 0, 0%
## low counts [2]     : 0, 0%
## (mean count < 3)
## [1] see 'cooksCutoff' argument of ?results
## [2] see 'independentFiltering' argument of ?results
DESeq2::plotMA(res,ylim=c(-10,10))


We now output the results as a *GRanges* object, and due to the conventions of *plyranges*, we construct a new column called gene_id from the row names of the results. Each row now contains the genomic region ( seqnames, start, end, strand) along with corresponding metadata columns (the gene_id and the results of the test). Note that *tximeta* has correctly identified the reference genome as “hg38”, and this has also been added to the *GRanges* along the results columns. This kind of book-keeping is vital once overlap operations are performed to ensure that *plyranges* is not comparing across incompatible genomes.


suppressPackageStartupMessages(library(plyranges))
de_genes <-results(dds,
contrast=c("condition","IFNg","naive"),
lfcThreshold=1,
format="GRanges")%>%
names_to_column("gene_id")
de_genes
## GRanges object with 17806 ranges and 7 metadata columns:
##           seqnames              ranges strand |            gene_id
##              <Rle>           <IRanges>  <Rle> |        <character>
##       [1]     chrX 100627109-100639991      - | ENSG00000000003.14
##       [2]    chr20   50934867-50958555      - | ENSG00000000419.12
##       [3]     chr1 169849631-169894267      - | ENSG00000000457.13
##       [4]     chr1 169662007-169854080      + | ENSG00000000460.16
##       [5]     chr1   27612064-27635277      - | ENSG00000000938.12
##       ...      ...                 ...    ... .                ...
##   [17802]    chr10   84167228-84172093      - |  ENSG00000285972.1
##   [17803]     chr6   63572012-63583587      + |  ENSG00000285976.1
##   [17804]    chr16   57177349-57181390      + |  ENSG00000285979.1
##   [17805]     chr8 103398658-103501895      - |  ENSG00000285982.1
##   [17806]    chr10   12563151-12567351      + |  ENSG00000285994.1
##                   baseMean     log2FoldChange              lfcSE
##                  <numeric>          <numeric>          <numeric>
##       [1] 171.570646163445 -0.282245015065582  0.300571026277417
##       [2] 967.751278980391 0.0391222756936352 0.0859707605047955
##       [3] 682.432885098654    1.2846178585311  0.196906721741941
##       [4] 262.963397841117  -1.47187616421189  0.218691645887265
##       [5] 2660.10225731917  0.675478091290521  0.236053041372838
##       ...              ...                ...                ...
##   [17802] 10.0474624496157  0.548451844773876  0.444318686394084
##   [17803] 4586.34616821518 -0.033929582570062  0.188004977365846
##   [17804] 14.2965310090402  0.312347650582085  0.522699844356108
##   [17805] 27.7629588245413  0.994518742790125   1.58237312176743
##   [17806] 6.60408582708505   0.25399752352481    0.5957511892896
##                        stat             pvalue              padj
##                   <numeric>          <numeric>         <numeric>
##       [1]                 0                  1                 1
##       [2]                 0                  1                 1
##       [3]  1.44544511235177  0.148332899695748                 1
##       [4] -2.15772377722715 0.0309493141635637 0.409727500369082
##       [5]                 0                  1                 1
##       ...               ...                ...               ...
##   [17802]                 0                  1                 1
##   [17803]                 0                  1                 1
##   [17804]                 0                  1                 1
##   [17805]                 0                  1                 1
##   [17806]                 0                  1                 1
##   -------
##   seqinfo: 25 sequences (1 circular) from hg38 genome


From this, we can restrict the results to those that meet our FDR threshold and select (and rename) the metadata columns we’re interested in:


de_genes <- de_genes%>%
  filter(padj<0.01)%>%
  select(gene_id,de_log2FC =log2FoldChange,de_padj =padj)



We now wish to extract genes for which there is evidence that the LFC is *not* large. We perform this test by specifying an LFC threshold and an alternative hypothesis ( altHypothesis) that the LFC is less than the threshold in absolute value. To visualize the result of this test, you can run results without format="GRanges", and pass this object to plotMA as before.

We label these genes as other_genes and later as “non-DE genes”, for comparison with our de_genes set.


other_genes <-results(dds,
contrast=c(
                        "condition","IFNg","naive"),
lfcThreshold=1,
altHypothesis="lessAbs",
format="GRanges")%>%
filter(padj<0.01)%>%
names_to_column("gene_id")%>%
dplyr::select(gene_id,
de_log2FC =log2FoldChange,
de_padj =padj)


### ATAC-seq peak differential abundance analysis

The following section describes the process we have used for generating a *GRanges* object of differential peaks from the ATAC-seq data in Alasoo *et al.* (2018).

The code chunks for the remainder of this section are not run.

For assessing differential accessibility, we run *limma* ( Smyth, 2004), and generate the a summary of LFCs and adjusted p-values for the peaks:


library(limma)
design <-model.matrix(~donor+condition,colData(atac))
fit <-lmFit(assay(atac), design)
fit <-eBayes(fit)
idx <-which(colnames(fit$coefficients)=="conditionIFNg")
tt <-topTable(fit,coef=idx,sort.by="none",n=nrow(atac))


We now take the rowRanges of the *SummarizedExperiment* and attach the LFCs and adjusted p-values from *limma*, so that we can consider the overlap with differential expression. Note that we set the genome build to “hg38” and restyle the chromosome information to use the “UCSC” style (e.g. “chr1”, “chr2”, etc.). Again, we know the genome build from the Zenodo entry for the ATAC-seq data.


atac_peaks <-rowRanges(atac)%>%
remove_names()%>%
mutate(
da_log2FC =tt$logFC,
da_padj =tt$adj.P.Val
)%>%
set_genome_info(genome ="hg38")

seqlevelsStyle(atac_peaks) <-"UCSC"


The final *GRanges* object containing the DA peaks is included in the workflow package and can be loaded as follows:


library(fluentGenomics)
peaks
## GRanges object with 296220 ranges and 3 metadata columns:
##            seqnames              ranges strand |          peak_id
##               <Rle>           <IRanges>  <Rle> |      <character>
##        [1]     chr1          9979-10668      * |      ATAC_peak_1
##        [2]     chr1         10939-11473      * |      ATAC_peak_2
##        [3]     chr1         15505-15729      * |      ATAC_peak_3
##        [4]     chr1         21148-21481      * |      ATAC_peak_4
##        [5]     chr1         21864-22067      * |      ATAC_peak_5
##        ...      ...                 ...    ... .              ...
##   [296216]     chrX 155896572-155896835      * | ATAC_peak_296216
##   [296217]     chrX 155958507-155958646      * | ATAC_peak_296217
##   [296218]     chrX 156016760-156016975      * | ATAC_peak_296218
##   [296219]     chrX 156028551-156029422      * | ATAC_peak_296219
##   [296220]     chrX 156030135-156030785      * | ATAC_peak_296220
##                     da_log2FC              da_padj
##                     <numeric>            <numeric>
##        [1]  0.266185396736073 9.10672732956434e-05
##        [2]   0.32217712436691 2.03434717570469e-05
##        [3] -0.574159538548115 3.41707743345703e-08
##        [4]  -1.14706617895329 8.22298606986521e-26
##        [5] -0.896143162633654 4.79452571676397e-11
##        ...                ...                  ...
##   [296216] -0.834628897017445  1.3354605397165e-11
##   [296217] -0.147537281935847    0.313014754316915
##   [296218] -0.609732301631964 3.62338775135558e-09
##   [296219] -0.347678474957794 6.94823191242968e-06
##   [296220]  0.492442459200901 7.07663984067763e-13
##   -------
##   seqinfo: 23 sequences from hg38 genome; no seqlengths


## Integrate ranges

### Finding overlaps with *plyranges*

We have already used *plyranges* a number of times above, to filter, mutate, and select on *GRanges* objects, as well as ensuring the correct genome annotation and style has been used. The *plyranges* package provides a grammar for performing transformations of genomic data ( Lee *et al.*, 2019). Computations resulting from compositions of *plyranges* “verbs” are performed using underlying, highly optimized range operations in the *GenomicRanges* package ( Lawrence *et al.* 2013).

For the overlap analysis, we filter the annotated peaks to have a nominal FDR bound of 1%.


da_peaks <- peaks%>%
filter(da_padj<0.01)


We now have *GRanges* objects that contain DE genes, genes without strong signal of DE, and DA peaks. We are ready to answer the question: is there an enrichment of DA ATAC-seq peaks in the vicinity of DE genes compared to genes without sufficient DE signal?

### Down sampling non-differentially expressed genes

As *plyranges* is built on top of *dplyr*, it implements methods for many of its verbs for *GRanges* objects. Here we can use slice to randomly sample the rows of the other_genes. The sample.int function will generate random samples of size equal to the number of DE-genes from the number of rows in other_genes:


size <-length(de_genes)
slice(other_genes,sample.int(n(), size))
## GRanges object with 749 ranges and 3 metadata columns:
##         seqnames              ranges strand |            gene_id
##            <Rle>           <IRanges>  <Rle> |        <character>
##     [1]     chr1   26890488-26900466      - | ENSG00000198746.12
##     [2]     chr4 141220887-141234697      + | ENSG00000109445.10
##     [3]    chr12 112160188-112382439      - | ENSG00000173064.12
##     [4]    chr13   31134974-31162388      - | ENSG00000120694.19
##     [5]    chr17   37514797-37609496      - |  ENSG00000275066.4
##     ...      ...                 ...    ... .                ...
##   [745]    chr20     3045945-3048254      + |  ENSG00000125901.5
##   [746]    chr16   70346829-70373383      + | ENSG00000168872.16
##   [747]    chr19   18831938-18868236      + | ENSG00000005007.12
##   [748]    chr12     6666477-6689572      - | ENSG00000126746.17
##   [749]     chr2     3575205-3580920      + | ENSG00000171863.14
##                   de_log2FC              de_padj
##                   <numeric>            <numeric>
##     [1]    0.16909882503824 1.36439677663303e-15
##     [2]  -0.110147580079407 2.08542530094741e-11
##     [3]   0.144029835606733 1.68431882130248e-07
##     [4]  -0.023454391472986 0.000530800735408018
##     [5]   0.252722313137111  2.0839123931282e-11
##     ...                 ...                  ...
##   [745]  -0.424589720947925  0.00440766986950405
##   [746]  0.0726651236153919 2.26389160906564e-09
##   [747] -0.0847460249226525  7.4089673378162e-29
##   [748]  0.0158683098473536 1.15330450566857e-13
##   [749]  -0.416796080579922 1.83298968911328e-08
##   -------
##   seqinfo: 25 sequences (1 circular) from hg38 genome


We can repeat this many times to create many samples via replicate. By replicating the sub-sampling multiple times, we minimize the variance on the enrichment statistics induced by the sampling process.


# set a seed for the results
set.seed(2019-08-02)
boot_genes <-replicate(
                        10,
                          
                        slice(other_genes,sample.int(
                        n(), size)),
simplify =FALSE)


This creates a list of *GRanges* objects as a list, and we can bind these together using the bind_ranges function. This function creates a new column called “resample” on the result that identifies each of the input *GRanges* objects:


boot_genes <-bind_ranges(boot_genes,.id ="resample")


Similarly, we can then combine the boot_genes *GRanges*, with the DE *GRanges* object. As the resample column was not present on the DE *GRanges* object, this is given a missing value which we recode to a 0 using mutate()


all_genes <-bind_ranges(
de=de_genes,
not_de = boot_genes,
  
                        .id="origin"
)%>%
mutate(
origin =factor(origin,c("not_de", "de")),
resample =ifelse(
                        is.na(resample), 0L,as.integer(resample))
  )
all_genes

## GRanges object with 8239 ranges and 5 metadata columns:
##          seqnames              ranges strand |            gene_id
##             <Rle>           <IRanges>  <Rle> |        <character>
##      [1]     chr1 196651878-196747504      + | ENSG00000000971.15
##      [2]     chr6   46129993-46146699      + |  ENSG00000001561.6
##      [3]     chr4   17577192-17607972      + | ENSG00000002549.12
##      [4]     chr7 150800403-150805120      + |  ENSG00000002933.8
##      [5]     chr4   15778275-15853230      + | ENSG00000004468.12
##      ...      ...                 ...    ... .                ...
##   [8235]    chr17   43527844-43579620      - | ENSG00000175832.12
##   [8236]    chr17   18260534-18266552      + | ENSG00000177427.12
##   [8237]    chr20   63895182-63936031      + | ENSG00000101152.10
##   [8238]     chr1   39081316-39487177      + | ENSG00000127603.25
##   [8239]     chr8   41577187-41625001      + | ENSG00000158669.11
##                   de_log2FC              de_padj  resample   origin
##                   <numeric>            <numeric> <integer> <factor>
##      [1]   4.98711071930695 1.37057050625117e-13         0       de
##      [2]   1.92721595378787  3.1747750217733e-05         0       de
##      [3]   2.93372501059128  2.0131038573066e-11         0       de
##      [4]   3.16721751137972 1.07359906028984e-08         0       de
##      [5]   5.40894352968188 4.82904694023763e-18         0       de
##      ...                ...                  ...       ...      ...
##   [8235] -0.240918426099239  0.00991611085813261        10   not_de
##   [8236] -0.166059030395757  9.1205141062356e-05        10   not_de
##   [8237]  0.250538999517482 1.74084544559733e-09        10   not_de
##   [8238] -0.385053503003028  0.00265539384929076        10   not_de
##   [8239]  0.155922038318879  2.9637514745875e-17        10   not_de
##   -------
##   seqinfo: 25 sequences (1 circular) from hg38 genome


### Expanding genomic coordinates around the transcription start site

Now we would like to modify our gene ranges so they contain the 10 kilobases on either side of their transcription start site (TSS). There are many ways one could do this, but we prefer an approach via the anchoring methods in *plyranges*. Because there is a mutual dependence between the start, end, width, and strand of a *GRanges* object, we define anchors to fix one of start and end, while modifying the width. As an example, to extract just the TSS, we can anchor by the 5’ end of the range and modify the width of the range to equal 1.


all_genes <- all_genes %>%
anchor_5p()%>%
mutate(width =1)


Anchoring by the 5’ end of a range will fix the end of negatively stranded ranges, and fix the start of positively stranded ranges.

We can then repeat the same pattern but this time using anchor_center() to tell *plyranges* that we are making the TSS the midpoint of a range that has total width of 20 kb, or 10 kb both upstream and downstream of the TSS.


all_genes <- all_genes%>%
anchor_center()%>%
mutate(width=2*1e4)


### Use overlap joins to find relative enrichment

We are now ready to compute overlaps between RNA-seq genes (our DE set and bootstrap sets) and the ATAC-seq peaks. In *plyranges*, overlaps are defined as joins between two *GRanges* objects: a *left* and a *right GRanges* object. In an overlap join, a match is any range on the *left GRanges* that is overlapped by the *right GRanges*. One powerful aspect of the overlap joins is that the result maintains all (metadata) columns from each of the *left* and *right* ranges which makes downstream summaries easy to compute.

To combine the DE genes with the DA peaks, we perform a left overlap join. This returns to us the all_genes ranges (potentially with duplication), but with the metadata columns from those overlapping DA peaks. For any gene that has no overlaps, the DA peak columns will have NA’s.


genes_olap_peaks <- all_genes%>%
join_overlap_left(da_peaks)
genes_olap_peaks

## GRanges object with 27766 ranges and 8 metadata columns:
##           seqnames              ranges strand |            gene_id
##              <Rle>           <IRanges>  <Rle> |        <character>
##       [1]     chr1 196641878-196661877      + | ENSG00000000971.15
##       [2]     chr6  46119993-46139992       + |  ENSG00000001561.6
##       [3]     chr4  17567192-17587191       + | ENSG00000002549.12
##       [4]     chr4  17567192-17587191       + | ENSG00000002549.12
##       [5]     chr4  17567192-17587191       + | ENSG00000002549.12
##       ...      ...                ...     ... .                ...
##   [27762]     chr1  39071316-39091315       + | ENSG00000127603.25
##   [27763]     chr1  39071316-39091315       + | ENSG00000127603.25
##   [27764]     chr8  41567187-41587186       + | ENSG00000158669.11
##   [27765]     chr8  41567187-41587186       + | ENSG00000158669.11
##   [27766]     chr8  41567187-41587186       + | ENSG00000158669.11
##                   de_log2FC               de_padj  resample   origin
##                   <numeric>             <numeric> <integer> <factor>
##       [1]  4.98711071930695  1.37057050625117e-13         0       de
##       [2]  1.92721595378787   3.1747750217733e-05         0       de
##       [3]  2.93372501059128   2.0131038573066e-11         0       de
##       [4]  2.93372501059128   2.0131038573066e-11         0       de
##       [5]  2.93372501059128   2.0131038573066e-11         0       de
##       ...               ...                   ...       ...      ...
##   [27762] -0.385053503003028  0.00265539384929076        10   not_de
##   [27763] -0.385053503003028  0.00265539384929076        10   not_de
##   [27764]  0.155922038318879  2.9637514745875e-17        10   not_de
##   [27765]  0.155922038318879  2.9637514745875e-17        10   not_de
##   [27766]  0.155922038318879  2.9637514745875e-17        10   not_de
##                    peak_id          da_log2FC              da_padj
##                <character>          <numeric>            <numeric>
##       [1]  ATAC_peak_21236 -0.546582189082724 0.000115273676444232
##       [2] ATAC_peak_231183   1.45329684862127  9.7322474682763e-17
##       [3] ATAC_peak_193578  0.222371496904895 3.00939005719989e-11
##       [4] ATAC_peak_193579 -0.281615137872819 7.99888515457195e-05
##       [5] ATAC_peak_193580  0.673705317951604 7.60042918890061e-15
##       ...              ...                ...                  ...
##   [27762]   ATAC_peak_5357  -1.05823584693303 3.69051674661467e-16
##   [27763]   ATAC_peak_5358  -1.31411238041643 6.44280493172654e-26
##   [27764] ATAC_peak_263396 -0.904080135059089 8.19576651692093e-13
##   [27765] ATAC_peak_263397  0.364737985368599 2.08834835864614e-08
##   [27766] ATAC_peak_263399  0.317386691052334 1.20088116314111e-08
##   -------
##   seqinfo: 25 sequences (1 circular) from hg38 genome


Now we can ask, how many DA peaks are near DE genes relative to “other” non-DE genes? A gene may appear more than once in genes_olap_peaks, because multiple peaks may overlap a single gene, or because we have re-sampled the same gene more than once, or a combination of these two cases.

For each gene (that is the combination of chromosome, the start, end, and strand), and the “origin” (DE vs not-DE) we can compute the distinct number of peaks for each gene and the maximum peak based on LFC. This is achieved via reduce_ranges_directed, which allows an aggregation to result in a *GRanges* object via merging neighboring genomic regions. The use of the directed suffix indicates we’re maintaining strand information. In this case, we are simply merging ranges (genes) via the groups we mentioned above. We also have to account for the number of resamples we have performed when counting if there are any peaks, to ensure we do not double count the same peak:


gene_peak_max_lfc <- genes_olap_peaks%>%
group_by(gene_id, origin)%>%
reduce_ranges_directed(
peak_count =sum(!is.na(da_padj))/n_distinct(resample),
peak_max_lfc =max(abs(da_log2FC))
)


**Figure 3.**
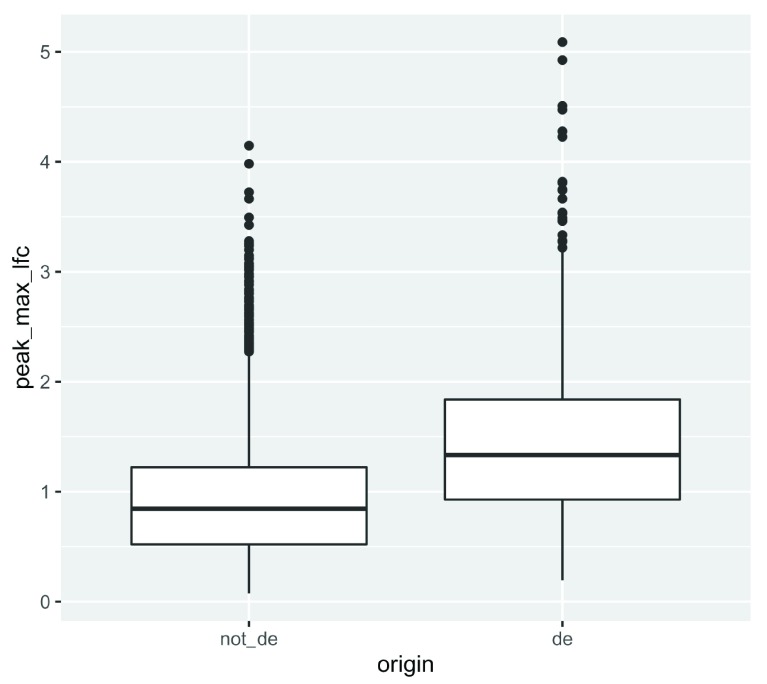
A boxplot of maximum LFCs for DA peaks for DE genes compared to non-DE genes where genes have at least one DA peak.

We can then filter genes if they have any peaks and compare the peak fold changes between non-DE and DE genes using a boxplot ( Figure 3):


library(ggplot2)
gene_peak_max_lfc%>%
filter(peak_count>0)%>%
as.data.frame()%>%
ggplot(aes(origin, peak_max_lfc))+
geom_boxplot()


In general, the DE genes have larger maximum DA fold changes relative to the non-DE genes.

Next we examine how thresholds on the DA LFC modify the enrichment we observe of DA peaks near DE or non-DE genes. First, we want to know how the number of peaks within DE genes and non-DE genes change as we change threshold values on the peak LFC. As an example, we could compute this by arbitrarily chosen LFC thresholds of 1 or 2 as follows:


origin_peak_lfc <- genes_olap_peaks%>%
group_by(origin)%>%
summarize(
peak_count =sum(!is.na(da_padj))/n_distinct(resample),
lfc1_peak_count =sum(abs(da_log2FC)>1,na.rm=TRUE)/n_distinct(resample),
lfc2_peak_count =sum(abs(da_log2FC)>2,na.rm=TRUE)/n_distinct(resample)
)
origin_peak_lfc

## DataFrame with 2 rows and 4 columns
##     origin peak_count lfc1_peak_count lfc2_peak_count
##   <factor>  <numeric>       <numeric>       <numeric>
## 1   not_de     2391.8           369.5            32.5
## 2       de       3416            1097             234


Here we see that DE genes tend to have more DA peaks near them, and that the number of DA peaks decreases as we increase the DA LFC threshold (as expected). We now show how to compute the ratio of peak counts from DE compared to non-DE genes, so we can see how this ratio changes for various DA LFC thresholds.

For all variables except for the origin column we divide the first row’s values by the second row, which will be the enrichment of peaks in DE genes compared to other genes. This requires us to reshape the summary table from long form back to wide form using the *tidyr* package. First, we pivot the results of the peak_count columns into name-value pairs, then pivot again to place values into the origin column. Then we create a new column with the relative enrichment:


origin_peak_lfc%>%
as.data.frame()%>%
tidyr::pivot_longer(cols =-origin)%>%
tidyr::pivot_wider(names_from =origin,values_from =value) %>%
  
                        mutate(enrichment =de/not_de)

## # A tibble: 3 x 4
##   name            not_de    de enrichment
##   <chr>            <dbl> <dbl>      <dbl>
## 1 peak_count      2392.   3416       1.43
## 2 lfc1_peak_count  370.   1097       2.97
## 3 lfc2_peak_count   32.5   234       7.2


The above table shows that relative enrichment increases for a larger LFC threshold.

Due to the one-to-many mappings of genes to peaks, it is unknown if we have the same number of DE genes participating or less, as we increase the threshold on the DA LFC. We can examine the number of genes with overlapping DA peaks at various thresholds by grouping and aggregating twice. First, the number of peaks that meet the thresholds are computed within each gene, origin, and resample group. Second, within the origin column, we compute the total number of peaks that meet the DA LFC threshold and the number of genes that have more than zero peaks (again averaging over the number of resamples).


genes_olap_peaks%>%
  group_by(gene_id, origin, resample)%>%
  reduce_ranges_directed(
    lfc1 =sum(abs(da_log2FC)>1,na.rm=TRUE),
    lfc2 =sum(abs(da_log2FC)>2,na.rm=TRUE)
  )%>%
  group_by(origin)%>%
  summarize(
    lfc1_gene_count =sum(lfc1>0)/n_distinct(resample),
    lfc1_peak_count =sum(lfc1)/n_distinct(resample),
    lfc2_gene_count =sum(lfc2>0)/n_distinct(resample),
    lfc2_peak_count =sum(lfc2)/n_distinct(resample)
  )

## DataFrame with 2 rows and 5 columns
##     origin lfc1_gene_count lfc1_peak_count lfc2_gene_count lfc2_peak_count
##   <factor>       <numeric>       <numeric>       <numeric>       <numeric>
## 1   not_de           271.2           369.5            30.3            32.5
## 2       de             515            1097             151             234


To do this for many thresholds is cumbersome and would create a lot of duplicate code. Instead we create a single function called count_above_threshold that accepts a variable and a vector of thresholds, and computes the sum of the absolute value of the variable for each element in the thresholds vector.


count_if_above_threshold <-function(var, thresholds) {
lapply(thresholds,function(.)sum(abs(var)>.,na.rm =TRUE))
}



The above function will compute the counts for any arbitrary threshold, so we can apply it over possible LFC thresholds of interest. We choose a grid of one hundred thresholds based on the range of absolute LFC values in the da_peaks *GRanges*

object:


thresholds <- da_peaks%>%
mutate(abs_lfc =abs(da_log2FC))%>%
with(
seq(min(abs_lfc),max(abs_lfc),length.out =100)
   )


The peak counts for each threshold are computed as a new list-column called value. First, the *GRanges* object has been grouped by the gene, origin, and the number of resamples columns. Then we aggregate over those columns, so each row will contain the peak counts for all of the thresholds for a gene, origin, and resample. We also maintain another list-column that contains the threshold values.


genes_peak_all_thresholds <- genes_olap_peaks%>%
group_by(gene_id, origin, resample)%>%
reduce_ranges_directed(
value =count_if_above_threshold(da_log2FC, thresholds),
threshold =list(thresholds)
)
genes_peak_all_thresholds
## GRanges object with 8239 ranges and 5 metadata columns:
##          seqnames              ranges strand |            gene_id   origin
##             <Rle>           <IRanges>  <Rle> |        <character> <factor>
##      [1]     chr1 196641878-196661877      + | ENSG00000000971.15       de
##      [2]     chr6   46119993-46139992      + |  ENSG00000001561.6       de
##      [3]     chr4   17567192-17587191      + | ENSG00000002549.12       de
##      [4]     chr7 150790403-150810402      + |  ENSG00000002933.8       de
##      [5]     chr4   15768275-15788274      + | ENSG00000004468.12       de
##      ...      ...                 ...    ... .                ...      ...
##   [8235]    chr17   43569620-43589619      - | ENSG00000175832.12   not_de
##   [8236]    chr17   18250534-18270533      + | ENSG00000177427.12   not_de
##   [8237]    chr20   63885182-63905181      + | ENSG00000101152.10   not_de
##   [8238]     chr1   39071316-39091315      + | ENSG00000127603.25   not_de
##   [8239]     chr8   41567187-41587186      + | ENSG00000158669.11   not_de
##           resample         value
##          <integer> <IntegerList>
##      [1]         0     1,1,1,...
##      [2]         0     1,1,1,...
##      [3]         0     6,6,6,...
##      [4]         0     4,4,4,...
##      [5]         0  11,11,11,...
##      ...       ...            ...
##   [8235]        10     1,1,1,...
##   [8236]        10     3,3,2,...
##   [8237]        10     5,5,5,...
##   [8238]        10     3,3,3,...
##   [8239]        10     3,3,3,...
##                                                           threshold
##                                                       <NumericList>
##      [1] 0.0658243106359027,0.118483961449043,0.171143612262182,...
##      [2] 0.0658243106359027,0.118483961449043,0.171143612262182,...
##      [3] 0.0658243106359027,0.118483961449043,0.171143612262182,...
##      [4] 0.0658243106359027,0.118483961449043,0.171143612262182,...
##      [5] 0.0658243106359027,0.118483961449043,0.171143612262182,...
##      ...                                                        ...
##   [8235] 0.0658243106359027,0.118483961449043,0.171143612262182,...
##   [8236] 0.0658243106359027,0.118483961449043,0.171143612262182,...
##   [8237] 0.0658243106359027,0.118483961449043,0.171143612262182,...
##   [8238] 0.0658243106359027,0.118483961449043,0.171143612262182,...
##   [8239] 0.0658243106359027,0.118483961449043,0.171143612262182,...
##   -------
##   seqinfo: 25 sequences (1 circular) from hg38 genome


Now we can expand these list-columns into a long *GRanges* object using the expand_ranges() function. This function will unlist the value and threshold columns and lengthen the resulting *GRanges* object. To compute the peak and gene counts for each threshold, we apply the same summarization as before:


origin_peak_all_thresholds <- genes_peak_all_thresholds%>%
  expand_ranges()%>%
  group_by(origin, threshold)%>%
  summarize(
    gene_count =sum(value>0)/n_distinct(resample),
    peak_count =sum(value)/n_distinct(resample)
  )
origin_peak_all_thresholds
## DataFrame with 200 rows and 4 columns
##       origin          threshold gene_count peak_count
##     <factor>          <numeric>  <numeric>  <numeric>
## 1     not_de 0.0658243106359027        708     2391.4
## 2     not_de  0.118483961449043      698.8     2320.6
## 3     not_de  0.171143612262182      686.2     2178.6
## 4     not_de  0.223803263075322      672.4     1989.4
## 5     not_de  0.276462913888462      650.4     1785.8
## ...      ...                ...        ...        ...
## 196       de   5.06849113788419          2          2
## 197       de   5.12115078869733          0          0
## 198       de   5.17381043951047          0          0
## 199       de   5.22647009032361          0          0
## 200       de   5.27912974113675          0          0


**Figure 4.**
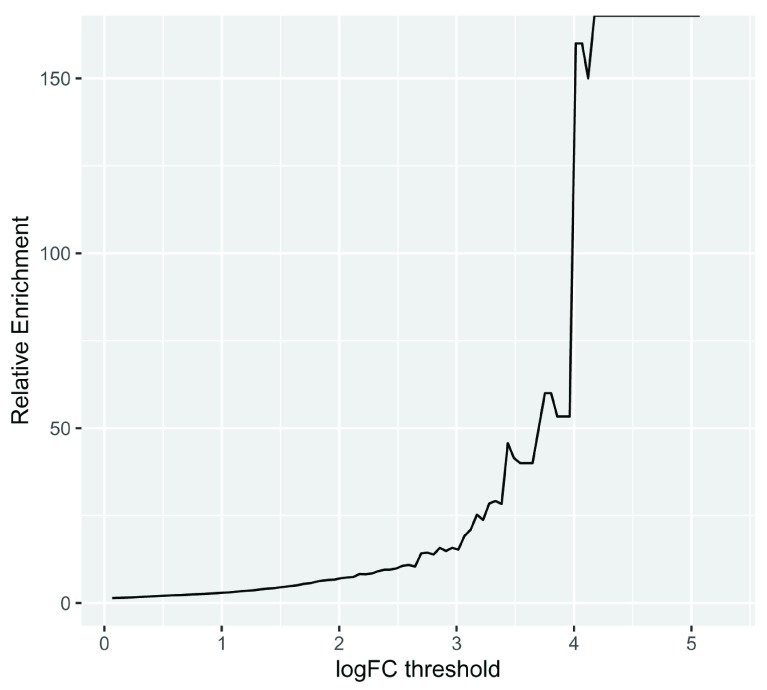
A line chart displaying how relative enrichment of DA peaks change between DE genes compared to non-DE genes as the absolute DA LFC threshold increases.

Again we can compute the relative enrichment in LFCs in the same manner as before, by pivoting the results to long form then back to wide form to compute the enrichment. We visualize the peak enrichment changes of DE genes relative to other genes as a line chart ( Figure 4):


origin_threshold_counts <- origin_peak_all_thresholds%>%
  as.data.frame()%>%
  tidyr::pivot_longer(cols =-c(origin, threshold),
                      names_to =c("type","var"),
                      names_sep ="_",
                      values_to ="count")%>%
  select(-var)

origin_threshold_counts%>%
  filter(type=="peak") 
                        %>%
  tidyr::pivot_wider(names_from =origin,values_from =count)%>%
  mutate(enrichment =de/not_de)%>%
  ggplot(aes(x =threshold,y =enrichment))+
  geom_line()+
  labs(x ="logFC threshold",y ="Relative Enrichment")

## Warning: Removed 4 row(s) containing missing values (geom_path).


**Figure 5.**
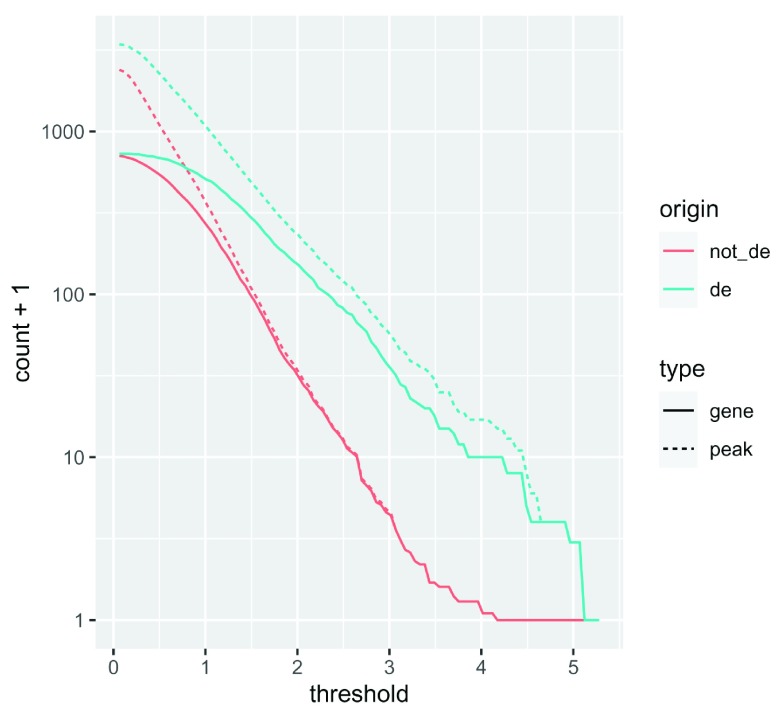
A line chart displaying how gene and peak counts change as the absolute DA LFC threshold increases. Lines are colored according to whether they represent a gene that is DE or not. Note the x-axis is on a log _10_ scale.

We computed the sum of DA peaks near the DE genes, for increasing LFC thresholds on the accessibility change. As we increased the threshold, the number of total peaks went down (likewise the mean number of DA peaks per gene). It is also likely the number of DE genes with a DA peak nearby with such a large change went down. We can investigate this with a plot ( Figure 5) that summarizes many of the aspects underlying the enrichment plot above.


origin_threshold_counts%>%
  ggplot(aes(x =threshold,             y =count+1,
             color =origin,
             linetype =type))+
  geom_line()+
  scale_y_log10()


## Discussion

We have shown that by using *plyranges* and *tximeta* (with support of Bioconductor and *tidyverse* ecosystems) we can fluently iterate through the biological data science workflow: from import, through to modeling, and data integration.

There are several further steps that would be interesting to perform in this analysis; for example, we could modify window size around the TSS to see how it affects enrichment, and vary the FDR cut-offs for both the DE gene and DA peak sets. We could also have computed variance in addition to the mean of the bootstrap set, and so drawn an interval around the enrichment line.

Finally, our workflow illustrates the benefits of using appropriate data abstractions provided by Bioconductor such as the *SummarizedExperiment* and *GRanges*. These abstractions provide users with a mental model of their experimental data and are the building blocks for constructing the modular and iterative analyses we have shown here. Consequently, we have been able to interoperate many decoupled R packages (from both Bioconductor and the tidyverse) to construct a seamless end-to-end workflow that is far too specialized for a single monolithic tool.

## Data availability

All data underlying the results are available as part of the article and no additional source data are required.

## Software availability

plyranges is available from Bioconductor: https://doi.org/doi:10.18129/B9.bioc.plyranges.

tximeta is available from Bioconductor: https://doi.org/doi:10.18129/B9.bioc.tximeta.

Source code and all workflow materials are available at: https://github.com/sa-lee/fluentGenomics.

Archived source code at time of publication: https://doi.org/10.5281/zenodo.3633505 ( Lee & Love, 2020).

License: MIT License.

The development version of the workflow and all downstream dependencies can be installed using the BiocManager package by running:


# development version from Github
BiocManager::install("sa-lee/fluentGenomics")
# version available from Bioconductor
BiocManager::install("fluentGenomics")


This article and the analyses were performed with R ( R Core Team, 2019) using the *rmarkdown* ( Allaire *et al.*, 2019), and *knitr* ( Xie, 2019; Xie, 2015) packages.

### Session Info


sessionInfo()
## R version 3.6.1 (2019-07-05)
## Platform: x86_64-apple-darwin15.6.0 (64-bit)
## Running under: macOS Mojave 10.14.6
##
## Matrix products: default
## BLAS:   /System/Library/Frameworks/Accelerate.framework/Versions/A/Frameworks/vecLib.framework/Versions/A/libBLAS.
## LAPACK: /Library/Frameworks/R.framework/Versions/3.6/Resources/lib/libRlapack.dylib
##
## locale:
## [1] en_AU.UTF-8/en_AU.UTF-8/en_AU.UTF-8/C/en_AU.UTF-8/en_AU.UTF-8
##
## attached base packages:
## [1] parallel  stats4    stats     graphics  grDevices utils     datasets
## [8] methods   base
##
## other attached packages:
##  [1] ggplot2_3.3.0.9000          plyranges_1.7.8
##  [3] DESeq2_1.26.0               GenomicFeatures_1.38.0
##  [5] AnnotationDbi_1.48.0        SummarizedExperiment_1.16.0
##  [7] DelayedArray_0.12.1         BiocParallel_1.20.0
##  [9] matrixStats_0.55.0          Biobase_2.46.0
## [11] GenomicRanges_1.38.0        GenomeInfoDb_1.22.0
## [13] IRanges_2.20.1              S4Vectors_0.24.1
## [15] BiocGenerics_0.32.0         readr_1.3.1
## [17] dplyr_0.8.3                 tximeta_1.4.2
## [19] fluentGenomics_0.0.5        rmarkdown_2.0
##
## loaded via a namespace (and not attached):
##   [1] colorspace_1.4-1         rprojroot_1.3-2          htmlTable_1.13.3
##   [4] XVector_0.26.0           base64enc_0.1-3          rstudioapi_0.10
##   [7] farver_2.0.3             bit64_0.9-7              fansi_0.4.1
##  [10] xml2_1.2.2               splines_3.6.1            tximport_1.14.0
##  [13] geneplotter_1.64.0       knitr_1.27               zeallot_0.1.0
##  [16] Formula_1.2-3            jsonlite_1.6             Rsamtools_2.2.1
##  [19] annotate_1.64.0          cluster_2.1.0            dbplyr_1.4.2
##  [22] png_0.1-7                compiler_3.6.1           httr_1.4.1
##  [25] backports_1.1.5          assertthat_0.2.1         Matrix_1.2-18
##  [28] lazyeval_0.2.2           cli_2.0.1                acepack_1.4.1
##  [31] htmltools_0.4.0          prettyunits_1.1.0        tools_3.6.1
##  [34] gtable_0.3.0             glue_1.3.1               GenomeInfoDbData_1.2.2
##  [37] rappdirs_0.3.1           Rcpp_1.0.3               vctrs_0.2.1
##  [40] Biostrings_2.54.0        rtracklayer_1.46.0       xfun_0.12
##  [43] stringr_1.4.0            lifecycle_0.1.0          ensembldb_2.10.2
##  [46] XML_3.99-0.3             zlibbioc_1.32.0          scales_1.1.0
##  [49] hms_0.5.3                ProtGenerics_1.18.0      AnnotationFilter_1.10.0
##  [52] RColorBrewer_1.1-2       yaml_2.2.0               curl_4.3
##  [55] memoise_1.1.0            gridExtra_2.3            biomaRt_2.42.0
##  [58] rpart_4.1-15             hunspell_3.0             latticeExtra_0.6-29
##  [61] stringi_1.4.5            RSQLite_2.2.0            genefilter_1.68.0
##  [64] checkmate_1.9.4          rlang_0.4.2              pkgconfig_2.0.3
##  [67] commonmark_1.7           bitops_1.0-6             evaluate_0.14
##  [70] lattice_0.20-38          purrr_0.3.3              labeling_0.3
##  [73] GenomicAlignments_1.22.1 htmlwidgets_1.5.1        bit_1.1-15.1
##  [76] tidyselect_0.2.5         here_0.1                 magrittr_1.5
##  [79] bookdown_0.16            R6_2.4.1                 spelling_2.1
##  [82] Hmisc_4.3-0              DBI_1.1.0                withr_2.1.2
##  [85] pillar_1.4.3             foreign_0.8-73           survival_3.1-8
##  [88] RCurl_1.98-1.1           nnet_7.3-12              tibble_2.1.3
##  [91] crayon_1.3.4             utf8_1.1.4               BiocFileCache_1.10.2
##  [94] jpeg_0.1-8.1             progress_1.2.2           locfit_1.5-9.1
##  [97] grid_3.6.1               data.table_1.12.8        blob_1.2.1
## [100] digest_0.6.23            xtable_1.8-4             tidyr_1.0.0
## [103] openssl_1.4.1            munsell_0.5.0            askpass_1.1

