# Supplementary material for: Fluent genomics with  plyranges and  tximeta
Source: F1000Res. 2020 Feb 12;9:109. [Version 1] doi: 10.12688/f1000research.22259.1 (PMC7243206; doi:10.12688/f1000research.22259.1)
Supplement: Peer review history

# Peer review: Reviewer response for version 1

**DOI:** 10.5256/f1000research.24553.r59959

**Published:** 2020-05-21

**Authors:** Michael M. Hoffman, Mickaël Mendez

**Affiliations:** 1 Department of Computer Science, University of Toronto, Toronto, ON, Canada; 2 Princess Margaret Cancer Centre, Toronto, ON, Canada; 3 Department of Medical Biophysics, University of Toronto, Toronto, ON, Canada; 4 Vector Institute, University of Toronto, Toronto, ON, Canada

The authors describe a workflow to perform exploratory analysis of biological data. This paper also highlights the importance of using tools that facilitate the application of essential concepts of exploratory data analysis: reproducibility and code readability.

 Major comments:

 1. I was not able to successfully complete the protocol myself, failing at the second step. I tried three different environments:

 - R 3.4.4 on Ubuntu 18.04.4 LTS/Windows Subsystem for Linux WSL1/Windows 10 1909 18363.836 (BiocManager refuses to install fluentGenomics on the system R for what is currently the latest version of Ubuntu available for general availability Windows users).

 - R 4.0.0 x86 on Windows 10 1909 18363.836

   - BiocManager::install("fluentGenomics")

   - BiocManager::install("sa-lee/fluentGenomics")

 R version 4.0.0 (2020-04-24) -- "Arbor Day"

 Copyright (C) 2020 The R Foundation for Statistical Computing

 Platform: i386-w64-mingw32/i386 (32-bit)

 R is free software and comes with ABSOLUTELY NO WARRANTY.

 You are welcome to redistribute it under certain conditions.

 Type 'license()' or 'licence()' for distribution details.

   Natural language support but running in an English locale

 R is a collaborative project with many contributors.

 Type 'contributors()' for more information and

 'citation()' on how to cite R or R packages in publications.

 Type 'demo()' for some demos, 'help()' for on-line help, or

 'help.start()' for an HTML browser interface to help.

 Type 'q()' to quit R.

 > library(fluentGenomics)

 > path_to_se <- cache_atac_se()

 Error in retrieve_cache() :

   Please install rappdirs to set cache directory

 > detach("package:fluentGenomics", unload=TRUE)

 > BiocManager::install("sa-lee/fluentGenomics")

 Bioconductor version 3.11 (BiocManager 1.30.10), R 4.0.0 (2020-04-24)

 Installing github package(s) 'sa-lee/fluentGenomics'

 Error: package 'remotes' not installed in library path(s)

     C:/Users/mhoffman/R/win-library/4.0

     C:/Program Files/R/R-4.0.0/library

 install with 'install("remotes")'

 > install.packages("remotes")

 […]

 > BiocManager::install("sa-lee/fluentGenomics")

 > path_to_se <- cache_atac_se()

 Error in retrieve_cache() :

   Please install rappdirs to set cache directory

 > sessionInfo()

 R version 4.0.0 (2020-04-24)

 Platform: i386-w64-mingw32/i386 (32-bit)

 Running under: Windows 10 x64 (build 18363)

 Matrix products: default

 locale:

 [1] LC_COLLATE=English_United States.1252  LC_CTYPE=English_United States.1252    LC_MONETARY=English_United States.1252

 [4] LC_NUMERIC=C                           LC_TIME=English_United States.1252    

 attached base packages:

 [1] stats     graphics  grDevices utils     datasets  methods   base    

 other attached packages:

 [1] fluentGenomics_0.99.3

 loaded via a namespace (and not attached):

  [1] Rcpp_1.0.4.6                BiocManager_1.30.10         compiler_4.0.0              pillar_1.4.4              

  [5] GenomeInfoDb_1.24.0         XVector_0.28.0              remotes_2.1.1               bitops_1.0-6              

  [9] tools_4.0.0                 zlibbioc_1.34.0             tibble_3.0.1                lifecycle_0.2.0            

 [13] lattice_0.20-41             pkgconfig_2.0.3             rlang_0.4.6                 Matrix_1.2-18              

 [17] DelayedArray_0.14.0         parallel_4.0.0              GenomeInfoDbData_1.2.3      rtracklayer_1.48.0        

 [21] dplyr_0.8.5                 hms_0.5.3                   plyranges_1.8.0             Biostrings_2.56.0          

 [25] S4Vectors_0.26.1            vctrs_0.3.0                 IRanges_2.22.1              stats4_4.0.0              

 [29] grid_4.0.0                  tidyselect_1.1.0            glue_1.4.1                  Biobase_2.48.0            

 [33] R6_2.4.1                    XML_3.99-0.3                BiocParallel_1.22.0         readr_1.3.1                

 [37] purrr_0.3.4                 magrittr_1.5                Rsamtools_2.4.0             matrixStats_0.56.0        

 [41] ellipsis_0.3.1              BiocGenerics_0.34.0         GenomicRanges_1.40.0        GenomicAlignments_1.24.0  

 [45] assertthat_0.2.1            SummarizedExperiment_1.18.1 RCurl_1.98-1.2              crayon_1.3.4              

 Minor comments:

 2. It is unclear who the audience for this paper is. As written it seems to presume a lot of technical knowledge about Bioconductor, R, and specific Bioconductor packages. It would not make a good starting point for someone who doesn’t have large familiarity with all of the above. This is too bad because it has the bones of something that could be useful to people with less expertise if some additional explanatory text were provided and clearer choices made about naming variables. There is also a danger that this will be used as a cookbook without understanding.

 I have marked some examples of things that could use more explanation but this is non-exhaustive. It would be worthwhile for the authors to examine the manuscript again and ask whether all of the large number of concepts, jargon, and names introduced are explained.

 3. The goal of the paper appeared to be clear after reading the paper. The introduction, figure 1, and following sections, however, leave the impression that the authors tried to reproduce results from another publication which is not the case. The paper would benefit from clarifying this toward the end of the introduction.

 4. Abstract: it is not clear which problem does the workflow solve and why is it important to solve it. For example, how does the import, model, and integrate structure increases result reproducibility? Is this structure new? Why would someone use it? We think that the abstract would benefit from more justification and moving the text specific to the tools used (tximeta, SummarizedExperiment) to the introduction.

 5. Abstract: The sentence “Using tximeta, ...” and the following can be confusing. Why is it only using of RNA-seq in the first sentence, and both RNA-seq and ATAC-seq in the following one?

 6. p3: Figure 1: The meaning of some of the objects is not immediately apparent. For example: coldata, se, dds, boot_genes. This figure does not make a great introduction for someone who is not already familiar with this workflow because so many of the details are mystifying. You should either simplify or explain more.

 7. p3: Figure 1: Text in boxes is blurry.

 8. p3: meaning of “fluent” should be described briefly here and not assumed

 9. Introduction: This section would benefit from a more thorough description of the tools used in the workflow and explain why they are important. For example: What is Bioconductor? Why is plyranges relevant for this analysis? What are tidyverse design principles and why are they relevant here?

 10. Introduction: Authors should also explain in the introduction why the “import, model, integrate” structure benefits  users.

 11. The authors should consistently use the same tense (we examine … we will …).

 12. Why ignoring genotypes?

 13. It would be useful to note that "genomic ranges" are often referred to as intervals elsewhere such as in BEDTools.

 14. Please cite Tximeta at the first occurrence.

 15. Experimental data: Here and in the following sections, the authors describe alternatively what the workflow does and what the workflow can do. For example: “The following code loads the path to the cached data file, or if it is not present, will create the cache and generate

 a SummarizedExperiment using”. We think that it would be clearer if these sections with code examples focus on what exactly the code does, like in a protocole paper. Describing what the tools can do should appear either in the introduction or in a separate paragraph. For the example above, the paragraph would explain how users would benefit from this behavior: save time?

 16. “A subset of the RNA-seq ...”, Why use only a subset?

 17. Typo: “be also be downloaded”

 18. p4: “GENCODE” not “Gencode”

 19. p4: Specify whether basic or comprehensive gene set used

 20. p4: Unclear what “Importing ATAC-seq data as a SummarizedExperiment object” refers to

 21. Import data as SummarizedExperiment: Authors should briefly describe what methods Soneson et al. use to summarize transcripts at gene level, and justify why they are doing it.

 22. p4: Unclear where the `coldata.csv` file is or what that name refers to. “file” should not be in monospace text

 23. p4: Placement of “Wickham et al., 2019” makes it appear to be a citation for R rather than tidyverse.

 24. p5: Many things here unclear to the uninitiated: what is the scary message when you load dplyr? What is a factor and relevel? What is a tibble? What do the ~ mean?

 25. p5: Meaning of “but would typically not have the ‘gz’ ending if used from Salmon directly” unclear

 26. p5: Should note the “files” column is going to be different for different users

 27. p6: What is suppressPackageStartupMessages?

 28. p6: What is a Salmon gtf?

 29. p6: Lots of unexplained details in the two transcripts on this page.

 30. p7: “tximeta knows the correct reference transcription”. How?

 31. p7: GRanges not described or cited

 32. p7: Unclear why only the start of + genes and not also their end is affected

 33. p7: The sentence starting “Another alternative would be” is complex and its meaning unclear (alternative to what?)

 34. Last paragraph of "Import data as SummarizedExperiment": authors describe alternative ways of summarizing genes and using their representative TSS. Is the point to explain that GRanges make these operations easier? We think a potential user would like to know how to perform precisely these operations. Otherwise move this to an earlier paragraph describing what the tool is capable of doing.

 35. p7: What does “109M” refer to? Please use SI or IEC units here and elsewhere

 36. p7: Should be “370 MB” or “370 MiB” not “~370 Mb”

 37. p7: It’s not clear how to actually get the tab-delimited files, a departure from what is otherwise detailed step-by-step instructions

 38. p7: Why skip=1? Why [,-1]?

 39. p7: It would be better if instead of just saying “we know this from the Zenodo entry” you instruct the workflow user to *check* the Zenodo entry to find out what genome assembly is used. A screenshot of that part of the Zenodo entry might be helpful.

 40. p7: Citation for Genome Reference Consortium needed

 41. p8: what is col_types? How do you know that this is what the col_types should be?

 42. p8: This is the first instance of a convention of using comments in monospace text for explanatory notes. I would avoid this and stick to notes in proportional text.

 43. p8: what is a “nominal” FDR?

 44. p8: greater than 1 or greater-than/equal? Suggest using > or >= for greater clarity

 45. Why 1%?

 46. p9: “MA” needs expansion, citation, and ideally brief explanation

 47. p9: Figure 2 caption has insufficient detail. Should describe what each point represents, and how many points there are. Should describe what method is used to adjust p-values. Please describe triangles versus circles. Explain the red horizontal line. Please indicate that “log fold change” means log2. Describe what “normalized counts are”

 48. p9: “due to the convention of plyranges”—meaning unclear

 49. p9: “This kind of book-keeping” = ?

 50. p9: Not everyone reading this will work on human genomics and know that hg38 = GRCh38

 51. p9: The authors should consistently put explanations of transcripts either below the transcript or above and not switch back and forth. (I think below is probably more helpful.)

 52. p10: please make clear that the altHypothesis here gets ALL of the other genes, that there are no problems with the edge case at LFC = threshold

 53. p11: Lots unexplained in the limma transcript

 54. p12: What is a “seqlength”?

 55. “genes without strong signal of DE”, are these “non-DE” genes? Would be better if used consistently.

 56. p12: Down sampling -> “Downsampling”

 57. p12: Unclear why one would want to downsample. The description only mentions that one can.

 58. p13: Please explain why one would want to set a seed.

 59. p13: “This creates a list of _GRanges_ objects as a list”. It would be clearer to say that this creates a _list_ of 10 _GRanges_ objects.

 60. What are "verbs" for GRanges

 61. p13: The importance of doing the bootstrap procedure here is really unclear

 62. p14: Why is there a circular sequence? I assume this is the mitochondrial chromosome? Does its presence in the background disturb the analysis at all?

 63. p14: "Now we would like to modify our gene ranges so they contain…" Why?

 64. p14: should be 5 prime not 5 apostrophe

 65. p15: the significance of the clarification that each “gene” is just a tuple of chromosome, start, end, strand is unclear

 66. p15: A lot of na.rm or is.na from here outward. Would be worth explaining why this is.

 67. p16: Figure 3: This figure needs descriptive labels instead of references to variables used in the code.

 68. p19: Explain why there are missing values

 69. p20: Should comment on why Figure 4 is non-monotonic

 70. p20: Saying the x-axis is on a log10 scale is a bit confusing because the y-axis is also on a log10 scale. It is better to make log scales more obvious by having non-uniform tick marks and grid lines. Also I don’t think the x-axis is on a log10 scale, log fold change here is measured with log2. (This would be more obvious if in the text you called it \log2 fold change consistently instead of the ambiguous “LFC”.)

 71. p21: It is unfortunate that the main point of this paper is to show that you can do something “fluently”, while what that means is never discussed or justified in the paper.

 72. p21: “There are several further steps that would be interesting to perform”, Are these steps easy to implement with the tools you described? If so, can you explain how easier it is with your approach?

 73. p21: “Finally, our workflow illustrates the benefits of using appropriate data abstractions”, This is probably obvious for R power users but not for other readers. Can you point at a specific section that shows that a user will benefit from using your approach?

 74. p21: The data availability statement is confusing. Clearly not all data are part of the article, they are part of external packages or articles (some specified without a particular URL or accession number, just reference to a paper).

 75. p21: instructions for installing BiocManager would be helpful, as would listing the *minimum* version of R or any other relevant packages. Also should mention that you need to library(BiocManager) first

 76. p21: It is unclear why you would want the development version, especially since it seems to be an older version number than what’s on Bioconductor

 77. p23: Items in the bibliography are not consistently referenced with specificity in a visible way. DOI should be provided for all Zenodo entries not just some. Some other documents that are not referenced via traditional publishing (journal volume and page number, or monograph from a publisher) should probably have DOI or URL specified also.

 Discretionary recommendations:

 78. p4: `path_to_se` is a poor name for a variable. It is not orthogonal to the other variable names used here and has little explanatory power. I recommend `atac_filename`

 79. p4: `dir` also is not descriptive. I recommend `quant_dirname`.

 80. p4: Would suggest more descriptive names than colfile/coldata, orthogonal to whatever naming scheme you use (which means either changes to names here or to use variations of the names I used above)

 81. p5: This transcript is a lot to digest, and it’s hard to know which parts of the explanatory text refer to which part of the transcript, which has multiple commands that go back a page previous. I suggest breaking it up more with explanatory text to the extent possible. Piping is convenient for a programmer but not for explanation. This comment applies to many of the subsequent transcripts too

 82. p5: Get the display of coldata so that it doesn’t exceed one line per row

 83. p6: It’s not a good idea to call your experiment object merely `se` when it is one of multiple SummarizedExperiments here

 84. p6: Please use a consistent style for spacing around = in R code

 85. p7: I suggest not giving the same name to different objects (`atac_mat` is first something like a data.frame and then a matrix.

 86. p7: Suggest not shadowing built-in functions with variable names like `rownames`.

 87. p7: “We know this” antecedent unclear

 88. p8: I suggest running all the R code in here through a linter. This will ensure you have consistent style.

 89. p8: res is not a specific variable name

 90. p10: Why switch between “other genes” and “non DE genes”? Just call them `not_de_genes` from the start. (Or switch to `genes_de` and `genes_not_de`.)

 91. p11: Why is library(fluentGenomics) loaded again?

 92. p11: Using library(fluentGenomics) adds a data object called `peaks` to your environment? This seems undesirable

 93. p12: The text would be a bit more readable if you eschewed the abbreviations “DA”, “DE”, and “LFC” in the main text.

 94. p13: here and elsewhere: integer literals (e.g. 10L) should be used instead of float literals for integers

 95. p14: why `2*1e4`? 20000L would be clearer

 96. p16: Figure 3: Please specify what the components of the box plot mean. There is no universal box plot.

Is the rationale for developing the new method (or application) clearly explained?

Partly

Is the description of the method technically sound?

Partly

Are the conclusions about the method and its performance adequately supported by the findings presented in the article?

Partly

If any results are presented, are all the source data underlying the results available to ensure full reproducibility?

No

Are sufficient details provided to allow replication of the method development and its use by others?

No

Reviewer Expertise:

computational genomics

We confirm that we have read this submission and believe that we have an appropriate level of expertise to confirm that it is of an acceptable scientific standard, however we have significant reservations, as outlined above.

# Peer review: Reviewer response for version 1

**DOI:** 10.5256/f1000research.24553.r61441

**Published:** 2020-04-30

**Authors:** Davis J. McCarthy, Ruqian Lyu, PuXue Qiao

**Affiliations:** 1 St Vincent’s Institute of Medical Research, Fitzroy, Victoria, Australia; 2 The University of Melbourne, Parkville, Victoria, Australia

Lee, Lawrence and Love present a workflow article about using a group of well-constructed Bioconductor packages for fluent genomic analysis that integrates both RNA-seq and ATAC-seq analysis results from the same samples (baseline and stimulus).

 The workflow provides instructions for obtaining differentially accessible (DA; peaks from ATAC-seq) and differential expression (DE; from RNA-seq) data objects, and more importantly a convenient way of integrating the ranged genomic features produced, while taking care of matching reference genome builds and other crucial “bookkeeping” tasks in the process.

 The authors have demonstrated analysis can be performed to check whether DA peaks are enriched in the vicinity of DE genes guided by the principle that transcriptomic response to IFNg stimulation may be mediated through binding of regulatory proteins to accessible regions, and these bindings can be detected by ATAC-seq if they increase the accessibility of these regions.

 The following steps are involved in the workflow:

 1. Downloading peaks data and RNA-seq data

 2. Perform DE and DA analysis

 3. Integrate ranges of DE and DA

 4. Test enrichment of DA in DE gene vicinity versus non-DE genes with various thresholds

**Major comments:**

1. The process of 'resampling' subsets of non-DE genes from the pool of non-DE genes should be clarified to explain the purpose of doing such 'resampling'. In particular, whether it can be considered 'bootstrapping' should be addressed. Our understanding is that the resampling here is done *without* *replacement* from DE results, whereas for bootstrapping we expect to sample data records *with replacement* and recompute statistics of interest from them.
2. Interpretation of the enrichment results or more discussion on the caveats of doing enrichment analysis like this are required.

**Minor comments:**

1. The workflow was built with R-3.6.1 which results in a discrepancy with the current package's requirement of R >= 4.0.0. However, this is understandable since we are near the new release of Bioconductor but be reminded to re-compile the workflow with released version.
2. A code chunk for checking that all required packages have been installed prior to other analysis in the environment would make the execution smoother for a user wanting to run the workflow.
3. The downloading function ` *cache_atac_se()`* raised an error when running on Windows. It can be fixed by setting mode = "wb" explicitly in the ` *download.file()* `.
4. Figure 1 which summaries the workflow could be improved by incorporating colored blocks for each package module and more descriptive elements companying the actual function calls or data objects. The caption also needs to provide more complete description of the elements of the figure for it to be comprehensible. These changes will give readers who are not familiar with all these packages more information.
5. The optional code chunks (such as the *Importing ATAC-seq data as a SummarizedExperiment object*) could be moved down to appendix to make it clearer to the readers which things are core parts of the workflow and which are included for full transparency/reproducibility/data provenance.
6. As for calculating the relative enrichment of peaks in DE versus non-DE, the description " *For all variables except for the origin column we divide the first row’s values by the second row*," was inaccurate and could corrected to "divide the DE row with the non-DE row"
7. It might worth emphasizing more on the contribution of this work in the abstract and introduction, maybe by stating explicitly the most attractive aspect of this work. For example, a guideline on integrating the ranged genomic features.
8. Make the sub-headings consistent. *Importing ATAC-seq, Import data.*
9. Some minor comments: *library(fluentGenomics)* appeared three times and *library(plyranges*) twice in the workflow. If this was to avoid namespace clashes, the function could be called explicitly *plyranges::* instead of loading the packages multiple times?
10. You could just use limma for the DE analysis as well and save yourself the loading of the DESeq2 package (joking!)

**Is the description of the method technically sound?**

 Partially. The “resampling” procedure needs a bit more explanation such as clarifying how and whether this is considered a bootstrapping method.

Is the rationale for developing the new method (or application) clearly explained?

Yes

Is the description of the method technically sound?

Partly

Are the conclusions about the method and its performance adequately supported by the findings presented in the article?

Yes

If any results are presented, are all the source data underlying the results available to ensure full reproducibility?

Yes

Are sufficient details provided to allow replication of the method development and its use by others?

Yes

Reviewer Expertise:

Statistics, bioinformatics, genomics, transcriptomics

We confirm that we have read this submission and believe that we have an appropriate level of expertise to confirm that it is of an acceptable scientific standard, however we have significant reservations, as outlined above.

# Peer review: Reviewer response for version 1

**DOI:** 10.5256/f1000research.24553.r60092

**Published:** 2020-03-10

**Authors:** Mark Dunning

**Affiliations:** 1 Sheffield Bioinformatics Core, University of Sheffield, Sheffield, UK

Lee et al. present a case study of using Bioconductor packages to recapitulate an existing analysis. The analysis is performed without the need to worry too much about genome versions and using tools from tidyverse which were previously incompatible with standard Bioconductor methodologies. I see this being a useful reference for experienced users on how to run a modern RNA-seq analysis and perform data integration.

 I am pleased to say that I could re-run the code and reproduce the results presented. I can think of a few potential changes to increase its utility for beginner Bioconductor users.

 The article assumes quite a lot of knowledge of tidyverse and GenomicRanges to understand the code chunks. Some pointers to where people can get more help on these packages would be useful. Some code chunks explicitly call methods from dplyr (dplyr::mutate), but not all. It would be good to be maintain consistency when calling dplyr methods, as the user would know where to get help.

 The authors go straight from creating the DESeq dataset to running the differential analysis. In reality, one would never do this without performing adequate QC checks. I'm sure the authors must have done this (as indeed the original study authors must have). But showing the code to generate a PCA would be useful; along with interpretation of the results

 The code is shown to remove genes with low counts prior to the DESeq analysis. I thought this was no longer necessary as DESeq2 incorporates it's own filtering. Or is this a memory-saving exercise? Some justification of why 10 counts and 6 samples was chosen would be beneficial to those new to Bioconductor and RNA-seq.

 The authors use Genomic ranges to facilitate the integration of different assay types. Whilst it is critical to keep the data in this form for analysis, ultimately collaborators will want the results in spreadsheet format with recognisable gene names. It would be useful if the authors could include an example code snippet to annotate the overlapping regions (e.g. with gene symbols) and exporting as a csv.

 It is my understanding that the authors present a simpler approach than that of Alasoo et al (2018) ^1^ in order to illustrate the utility of Bioconductor infrastructure. Nevertheless, some commentary on whether the same results and biological insights are revealed by their approach would be beneficial.

Is the rationale for developing the new method (or application) clearly explained?

Partly

Is the description of the method technically sound?

Yes

Are the conclusions about the method and its performance adequately supported by the findings presented in the article?

Yes

If any results are presented, are all the source data underlying the results available to ensure full reproducibility?

Yes

Are sufficient details provided to allow replication of the method development and its use by others?

Yes

Reviewer Expertise:

Bioinformatics, Bioconductor, Functional Genomics

I confirm that I have read this submission and believe that I have an appropriate level of expertise to confirm that it is of an acceptable scientific standard.

## References

[1] : Shared genetic effects on chromatin and gene expression indicate a role for enhancer priming in immune response. *Nat Genet*.50(3) : 10.1038/s41588-018-0046-7 424-431 10.1038/s41588-018-0046-7 29379200PMC6548559
